# Supplementary material for: PHD1 controls muscle mTORC1 in a hydroxylation-independent manner by stabilizing leucyl tRNA synthetase
Source: Nat Commun. 2020 Jan 10;11:174. doi: 10.1038/s41467-019-13889-6 (PMC6954236; doi:10.1038/s41467-019-13889-6)
Supplement: Supplementary file 1 — Supplementary information [file 41467_2019_13889_MOESM1_ESM.pdf]

SUPPLEMENTARY INFORMATION

PHD1 CONTROLS MUSCLE MTORC1 IN A HYDROXYLATION INDEPENDENT MANNER BY STABILIZING LEUCYL TRNA SYNTHETASE

D'Hulst G, Soro-Arnaiz I et al.

Supplementary Figure 1

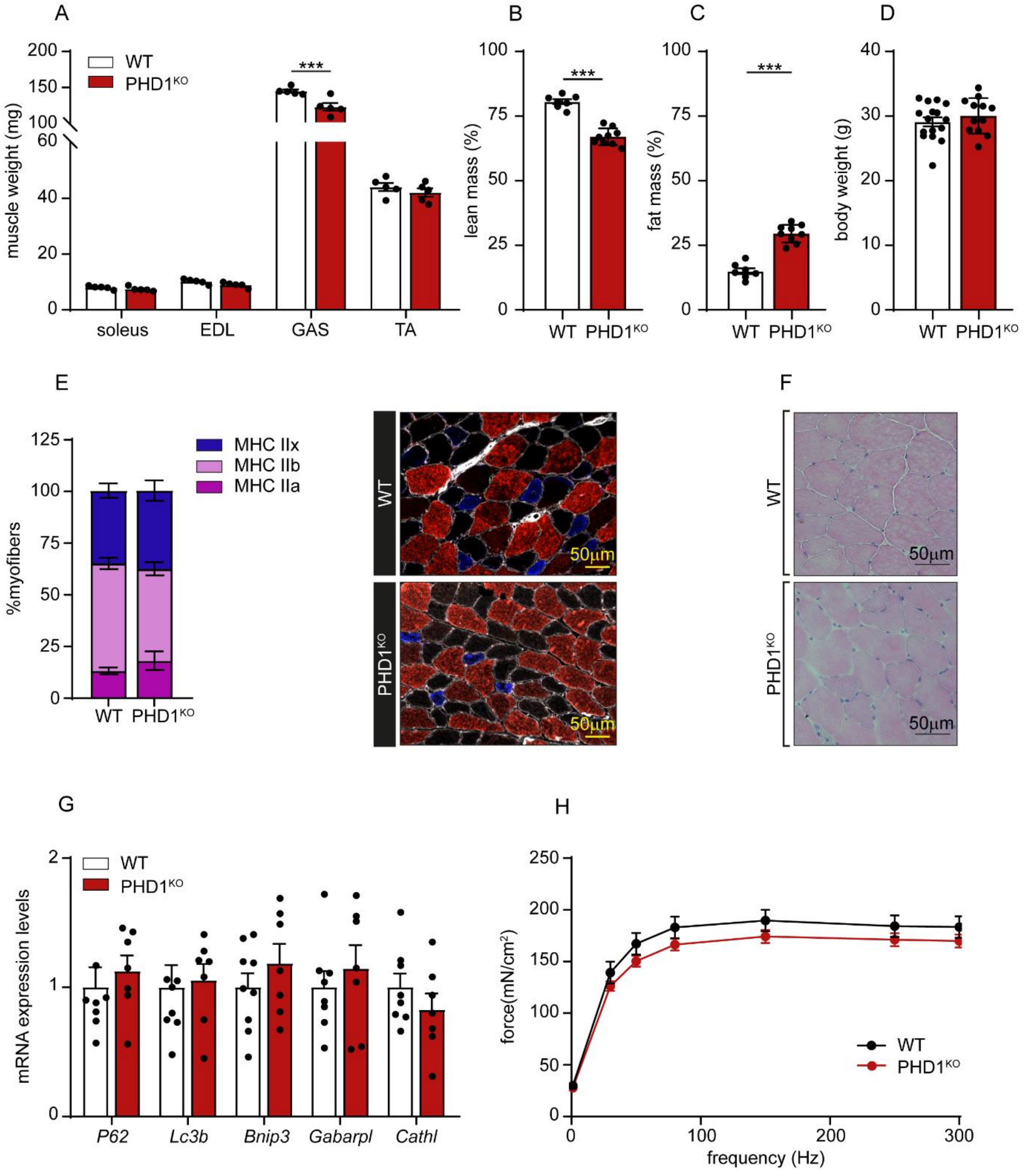

Supplementary Figure 1. Phd1 deficient mice have lower muscle mass. (Related to Figure 1)

Bar graphs showing muscle weight (A), lean mass (B), fat mass (C) and body weight (D) of WT (white bars) and PHD1^{KO} (red bars) female mice. (E) Quantification (left panel) and representative pictures (right panel) of fiber type distribution analysis in WT

(black line) and $PHD1^{KO}$ (red line) TA muscle (females). MHCIIa: blue, MHCIIb: red, MHCIIx: unstained, WGA: white. (F) Representative H&E pictures of TA muscle from WT and $PHD1^{KO}$ female mice. (G) mRNA expression levels of genes involved in the control of autophagy in TA muscle from WT (white bars) and $PHD1^{KO}$ (red bars) female mice. (H) Relative force–frequency curve in *ex-vivo* contracted *soleus* from WT (black line; n=9) and $PHD1^{KO}$ (red line; n=7) male mice. Force production (mN) is corrected for muscle surface area (cm^2).

Statistics: two-way ANOVA with a Holm-Sidak post-hoc test (H) or unpaired t test (A, B, C, D, E, G) (*p < 0.05; **p < 0.01; ***p < 0.001; ns, not significant). Each dot represents a single mouse. (A, B, C and D). Bars graphs and line graphs represent mean ± SEM (error bars). Data is represented as fold change to WT (G). Source data are provided as a Source Data file.

Supplementary Figure 2

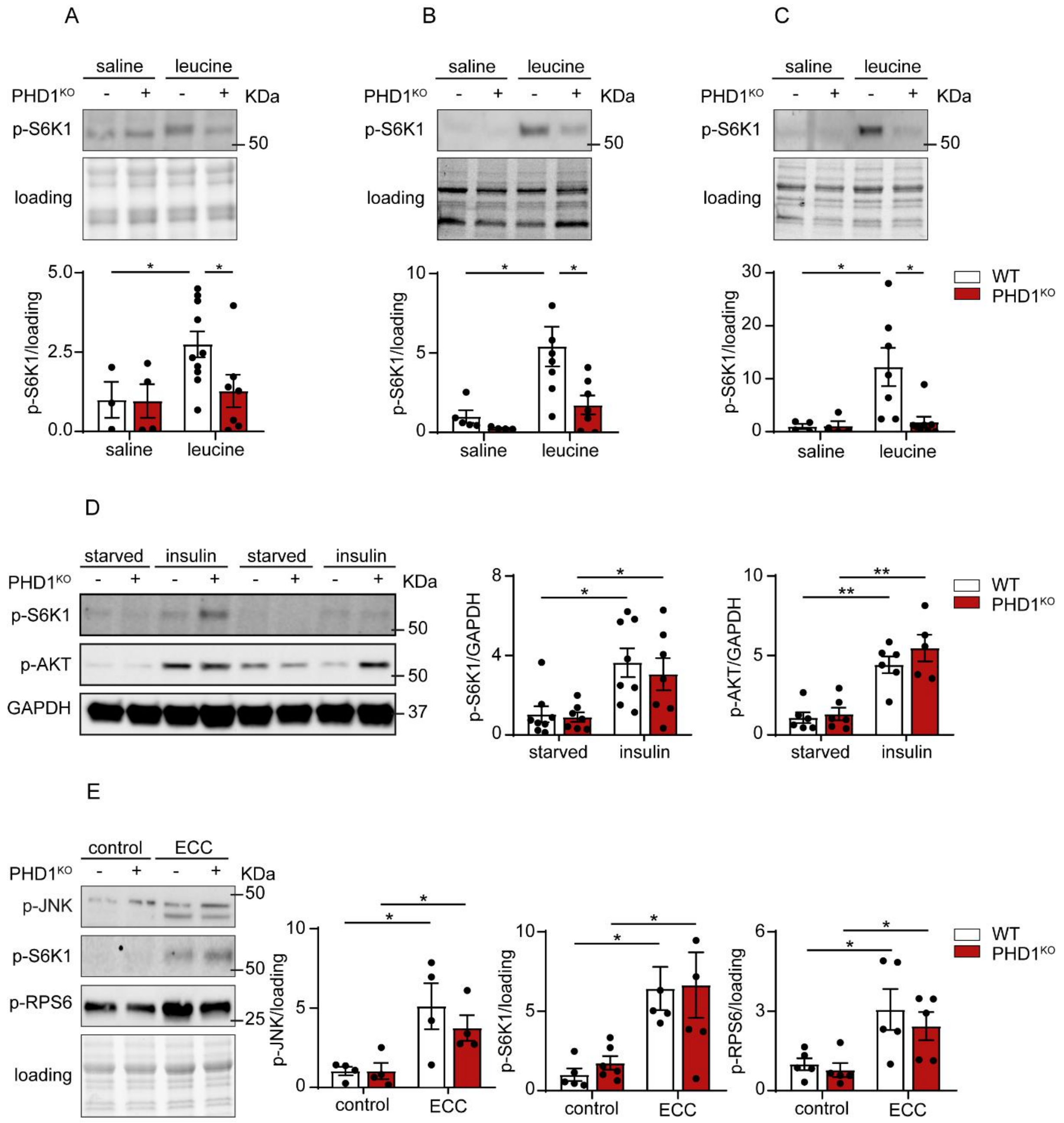

Supplementary Figure 2. PHD1 is not involved in insulin nor eccentric contraction-mediated mTORC1 activation *in vivo*. (Related to Figure 2)

(A) Representative pictures (top panel) and quantification (bottom panel) of western blot analysis of S6K1 phosphorylation in TA of male WT and PHD1^{KO}. Representative pictures (top panel) and quantification (bottom panel) of western blot analysis of S6K1 phosphorylation in *soleus* (B) and EDL (C) from WT (white bars) and PHD1^{KO} (red

bars) female mice 30 min after saline (saline) or leucine (leucine) gavage (D) Representative pictures (left panel) and quantification (right panels) of western blot analysis of S6K1 and AKT phosphorylation in TA muscles from WT (white bars) and $PHD1^{KO}$ (red bars) male mice 30 min after saline (starved) or insulin (insulin) injection. (E) Representative pictures (left panel) and quantification (right panels) of western blot analysis of S6K1, RPS6 and JNK phosphorylation in EDL from WT (white bars) and $PHD1^{KO}$ (red bars) male and female mice after an *ex vivo* eccentric contraction stimulation protocol (ECC) and unstimulated control (control). Source data are provided as a Source Data file.

Statistics: Mixed model two-way ANOVA test with a Holm-Sidak post-hoc test (A, B, C, D, E) (*p < 0.05; **p < 0.01; ***p < 0.001; ns, not significant). Each dot represents a single mouse. Bars graphs represent mean ± SEM (error bars). Data is presented as fold change to WT saline, starved or control. ECC: eccentric contractions. Source data are provided as a Source Data file.

Supplementary Figure 3

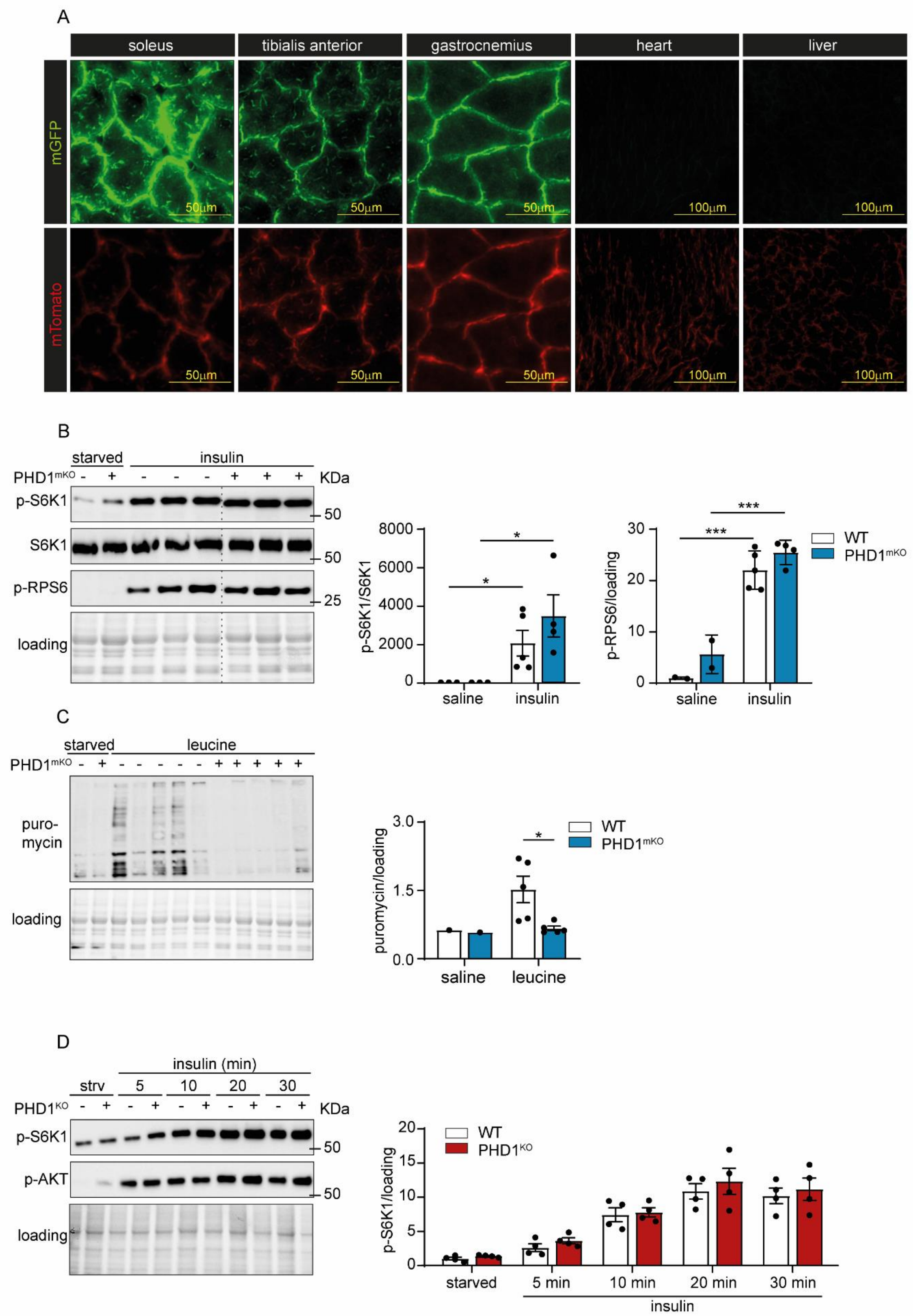

Supplementary Figure 3. Muscle specific loss of *Phd1* does not impair insulin-mediated mTORC1 activation. (Related to Figure 3)

(A) Representative pictures of different tissues isolated from HSA.iCre-mTmG mice 1 week after the last tamoxifen injection showing efficient recombination in skeletal muscles but not in other organs. (B) Representative pictures (left panel) and quantification (right panels) of western blot analysis of S6K1 and RPS6 phosphorylation in TA from WT (white bars) and $PHD1^{mKO}$ (blue bars) male and female mice 30 min after saline (saline) or insulin (insulin) injection. Each dot represents a single mouse. (C) Representative pictures (left panel) and quantification (right panel) of puromycin incorporation in TA muscle from WT and $PHD1^{mKO}$ male and female mice 30 min after leucine gavage. (D) Representative pictures (left panel) and quantification (right panel) of western blot analysis of S6K1 and AKT phosphorylation in differentiated myotubes isolated from WT (white bars) and $PHD1^{KO}$ (red bars) mice after 1h starvation (strv or starved) or at different time points after insulin stimulation (insulin). Dots represent experimental duplicates from 2 independent experiments.

Statistics: two-way ANOVA test with holm-sidak post-hoc test (B, D) and unpaired t test (C) (*$p < 0.05$; **$p < 0.01$; ***$p < 0.001$; ns, not significant). Bar graphs represent mean ± SEM (error bars). Data is presented as fold change to WT saline or starved. Source data are provided as a Source Data file.

Supplementary Figure 4

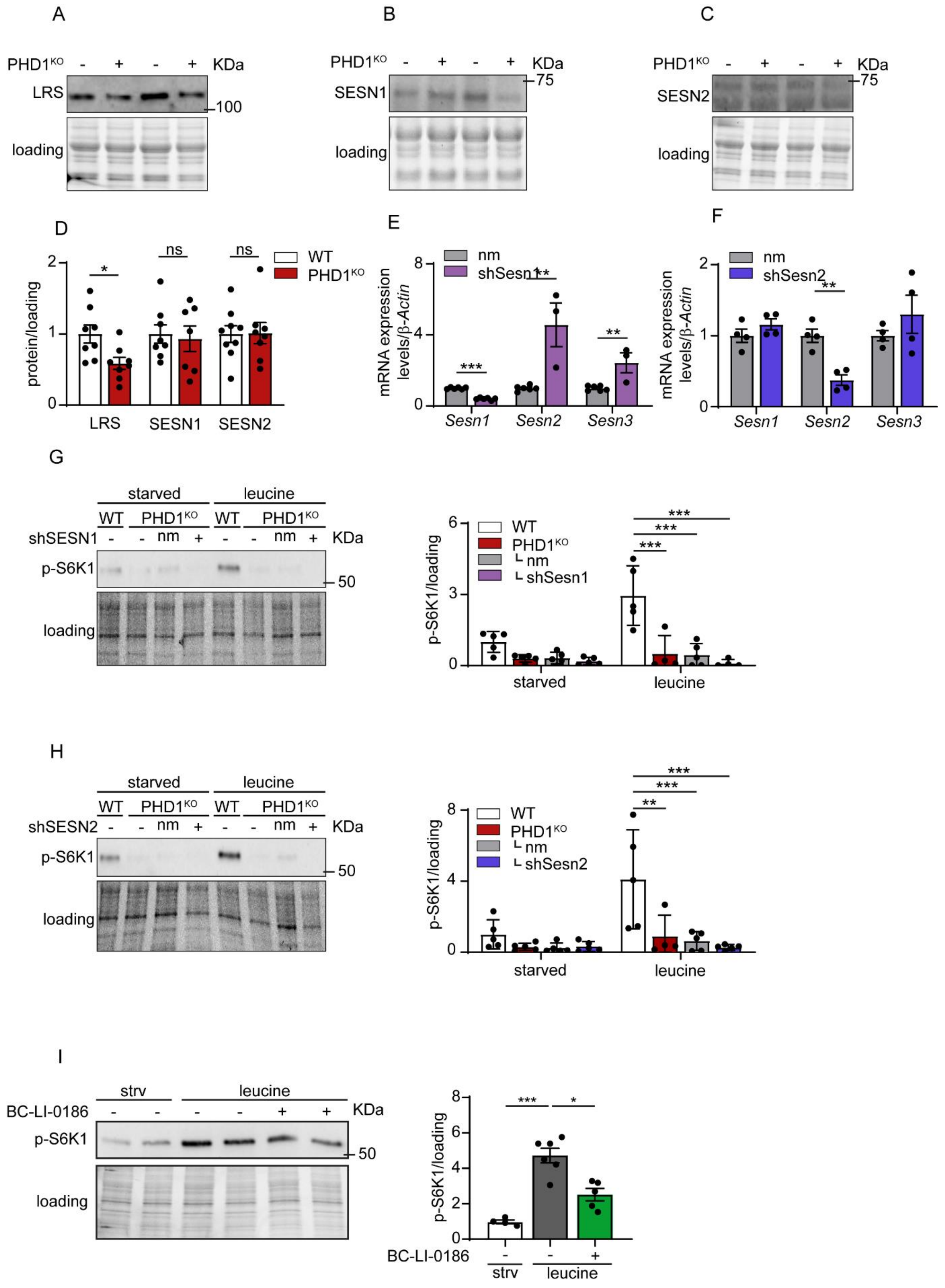

Supplementary Figure 4. PHD1 controls intracellular leucine sensing through leucyl tRNA synthetase. (Related to Figure 4)

Representative pictures (A-C) and quantification (D) of western blot analysis of LRS; SESN1-2 protein levels in TA muscles from WT (white bars) and PHD1^{KO} (red bars) female mice. mRNA expression of *Sesn1-3* in PHD1^{KO} myotubes with a short-hairpin for *Sesn1* (E) or *Sesn2* (F). (G-H) Representative picture (left panel) and quantification (right panel) of western blot analysis S6K1 phosphorylation in WT and PHD1^{KO} myotubes with a short-hairpin for *Sesn1* (G) or *Sesn2* (H) after 1 h starvation (starved) or 30 min stimulation with leucine (leucine). Dots represent replicates from 3 independent experiments. (I) Representative picture (left panel) and quantification (right panel) of western blot analysis of S6K1 phosphorylation in WT myotubes after 1 h starvation (starved, white bars) or 30 min stimulation with leucine (leucine) in the presence (green bars) or absence (grey bars) of the LRS inhibitor BC-LI-0186. Dots represent experimental duplicates from 3 independent experiments. Source data are provided as a Source Data file.

Statistics: one-way ANOVA test (I) or unpaired t test (D) (*$p < 0.05$; **$p < 0.01$; ***$p < 0.001$; ns, not significant). Each dot represents a single mouse (D). Bar graphs represent mean ± SEM (error bars). Source data are provided as a Source Data file

Supplementary Figure 5

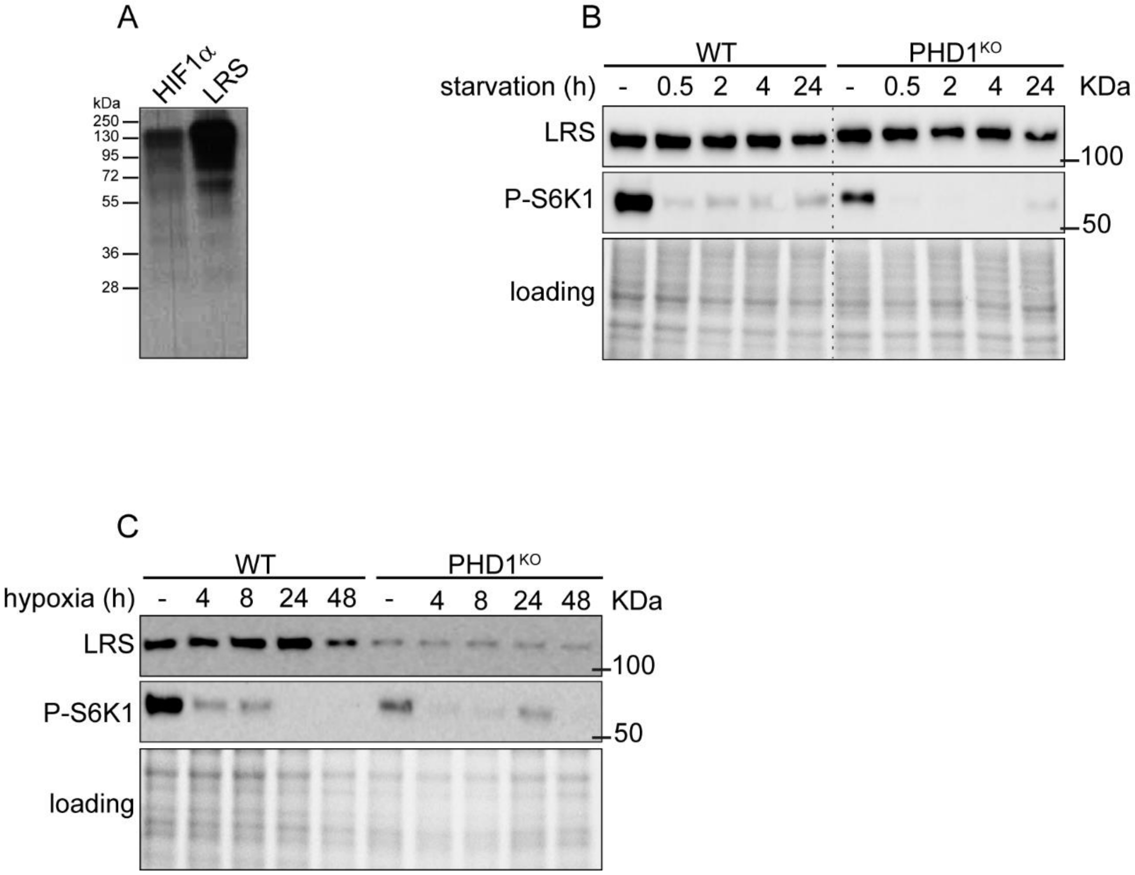

Supplementary Figure 5. PHD1 interacts with LRS and controls LRS stability. (Related to Figure 5)

(A) Fluorography showing efficient *in vitro* translation of HIF1α and LRS in the lysates that were used for *in vitro* hydroxylation assay (Figure 5B). (B) Representative western blot image of time-course analysis of LRS protein levels in WT and PHD1^{KO} myotubes exposed to amino acid starvation. (C) Representative western blot image of time-course analysis of LRS protein levels in LRS protein levels in WT and PHD1^{KO} myotubes exposed to hypoxia (1% oxygen). Source data are provided as a Source Data file.

Supplementary Table 1: Primary antibodies

4EBP1	Cell signaling	9452
LAMP2	Abcam	13524
LARS	Cell signaling	13868
LC3b	Novus Biologicals	NB100-222
mTOR	Cell signaling	2983
P62	Sigma-Aldrich	P0067
p-Akt^{Ser473}	Cell signaling	4060
PAN-leucylation	Home made	
p-mTOR^{Ser2448}	Cell signaling	5536
p-S6K1^{Thr389}	Cell signaling	9234
p-RPS6$^{Ser235/236}$	Cell signaling	2211
p-SAPK/JNK$^{thr183/tyr185}$	Cell signaling	9251
p-TSC2^{Ser1387}	Cell signaling	5584
Puromycin	Merk-millipore	MABE343
RagA 142 leucylation	Home made	
SESN2	Lubio	10795-1-AP
BA-F8 (MHCI)	Developmental Studies Hybridoma Bank	BA-F8
SC-71 (MHCIIa)	Developmental Studies Hybridoma Bank	SC-71
BF-F3 (MHCIIb)	Developmental Studies Hybridoma Bank	BF-F3
CD31-Alexa488	Biolegend	102414
CD45-Alexa488	Biolegend	103122
α7 INTEGRIN-PE	R&D Systems	FAB3518P
SCAI-APC	Biolegend	10811
PHD1	Novus Biologicals	NB100-310
p4EBP1Ser65	Cell signaling	9451

Supplementary Table 2: Secondary antibodies

Anti-Rabbit IgG	Cell signaling	7074
Anti-Mouse IgG	Cell signaling	7076
Goat anti-Mouse IgG2b AF 488	ThermoFisher Scientific	A-21141
Goat anti-Mouse IgG1 AF 350	ThermoFisher Scientific	A21120
Goat anti-Mouse IgM AF 568	ThermoFisher Scientific	A-21043

Supplementary Table 3: Primers

**Gene**	**Primer Fw**	**Primer Rv**
Phd1	CATCAATGGGCGCACCA	GATTGTCAACATGCCTCACGTAC
Phd2	AGCATACGCCACAAGGTACG	TACTTTAGCTCTCGCTCGCTC
Phd3	CAGACCGCAGGAATCCACAT	TTCAGCATCGAAGTACCAGACAGT
Lrs	AGCAGAGCACTGGCTTGATT	GCCGCCAGTCTACCTTCAAA
Gapdh	ACCCAGAAGACTGTGGATGG	CACATTGGGGGTAGGAACAC
B-actin	CTAAGGCCAACCGTGAAAAG	ACTTGTCGGAAGCCTCTTTG
Atrogin-1	TGGGTGTATCGGATGGAGAC	TCAGCCTCTGCATGATGTTC
Murf	TCCAGCAGACACTGAACCAGAA	TCCATTTTGCACCAATGTAGAAA
Itch	CCACCCACCCCACGAAGACC	CTAGGGCCCGAGCCTCCAGA
Smart	TCAATAACCTCAAGGCGTTC	GTTTTGCACACAAGCTCCA
Musa	TCGTGGAATGGTAATCTTGC	CCTCCCGTTTCTCTATCACG
FbxO31	GTATGGCGTTTGTGAGAACC	AGCCCCAAAATGTGTCTGTA
P62	CCCAGTGTCTTGGCATTCTT	AGGGAAAGCAGAGGAAGCTC
Bnip3	TAAACACCCGAA GCGCACA	GCCTTCCAATGTAGATCCCCA
Gabarapl	CATCGTGGAGAAGGCTCCTA	ATACAGCTGGCCCATGGTAG
Cathl	GTGGACTGTTCTCACGCTCAAG	TCCGTCCTTCGCTTCATAGG
Lc3b	CACTGCTCTGTCTTGTGTAGGTTG	TCGTTGTGCCTTTATTAGTGCATC
Lat1	CTTCGGCTCTGTCAATGGGT	TTCACCTTGATGGGACGCTC
Pat1	CCGCTACCATGTCCACACAG	GGCCACGATACCAATCACCA
Snat	GGCATTCAATAGCACCGCAG	ACGGAACTCCGGATAGGGAA
