# Supplementary material for: PHD1 controls muscle mTORC1 in a hydroxylation-independent manner by stabilizing leucyl tRNA synthetase
Source: Nat Commun. 2020 Jan 10;11:174. doi: 10.1038/s41467-019-13889-6 (PMC6954236; doi:10.1038/s41467-019-13889-6)
Supplement: Supplementary file 3 — Reporting summary [file 41467_2019_13889_MOESM3_ESM.pdf]

nature research

Corresponding author(s): Katrien De Bock
Last updated by author(s): Nov 15, 2019

Reporting Summary

Nature Research wishes to improve the reproducibility of the work that we publish. This form provides structure for consistency and transparency in reporting. For further information on Nature Research policies, see Authors & Referees and the Editorial Policy Checklist.

Statistics

For all statistical analyses, confirm that the following items are present in the figure legend, table legend, main text, or Methods section.

n/a	Confirmed	
☐	☒	The exact sample size (n) for each experimental group/condition, given as a discrete number and unit of measurement
☐	☒	A statement on whether measurements were taken from distinct samples or whether the same sample was measured repeatedly
☐	☒	The statistical test(s) used AND whether they are one- or two-sided<br>*Only common tests should be described solely by name; describe more complex techniques in the Methods section.*
☒	☐	A description of all covariates tested
☐	☒	A description of any assumptions or corrections, such as tests of normality and adjustment for multiple comparisons
☐	☒	A full description of the statistical parameters including central tendency (e.g. means) or other basic estimates (e.g. regression coefficient) AND variation (e.g. standard deviation) or associated estimates of uncertainty (e.g. confidence intervals)
☐	☒	For null hypothesis testing, the test statistic (e.g. F, t, r) with confidence intervals, effect sizes, degrees of freedom and P value noted<br>*Give P values as exact values whenever suitable.*
☒	☐	For Bayesian analysis, information on the choice of priors and Markov chain Monte Carlo settings
☒	☐	For hierarchical and complex designs, identification of the appropriate level for tests and full reporting of outcomes
☒	☐	Estimates of effect sizes (e.g. Cohen's d, Pearson's r), indicating how they were calculated

Our web collection on statistics for biologists contains articles on many of the points above.

Software and code

Policy information about availability of computer code

Data collection	Image J: plugin: Muscle J plugin (Mayeuf-Louchart et al, Skeletal muscle, 2018)<br>Image J: plugin: Colocalization plugin (Moser et al, Biotechnology journal, 2016)<br>Image Lab, Bio-Rad<br>Zen software (Zeiss)<br>Aurora software (Aurora Scientific)
Data analysis	Graphpad Prism 8.0

For manuscripts utilizing custom algorithms or software that are central to the research but not yet described in published literature, software must be made available to editors/reviewers. We strongly encourage code deposition in a community repository (e.g. GitHub). See the Nature Research guidelines for submitting code & software for further information.

Data

Policy information about availability of data

All manuscripts must include a data availability statement. This statement should provide the following information, where applicable:

- Accession codes, unique identifiers, or web links for publicly available datasets
- A list of figures that have associated raw data
- A description of any restrictions on data availability

We have added the following data availability statement to the manuscript:
The data presented in this study are available from the corresponding author upon reasonable request. The source data underlying Figs. 1-6 and Supplementary Figs. S1-5 are provided as a Source Data file

Field-specific reporting

Please select the one below that is the best fit for your research. If you are not sure, read the appropriate sections before making your selection.

☒ Life sciences ☐ Behavioural & social sciences ☐ Ecological, evolutionary & environmental sciences

For a reference copy of the document with all sections, see nature.com/documents/nr-reporting-summary-flat.pdf

Life sciences study design

All studies must disclose on these points even when the disclosure is negative.

Sample size	No sample-size calculations were performed. Sample size was determined to be adequate based on the magnitude and consistency of measurable differences between groups.
Data exclusions	Data that were deviating 4 SEM from the mean were excluded from analysis
Replication	At least 3 replicates were performed per experiment described in the experiment. Number of replicates are explained in the figure legends
Randomization	Mouse experiments: for mouse experiments with different treatments (leucine or saline injections) mice were randomly assigned to treatment groups.<br>Human experiments: subjects were assigned to each experimental group in a random order.
Blinding	Mouse experiments, investigators were blinded during the experiments as well as during post mortem tissue analysis. Cell experiments, investigators were not blinded to cell genotype during preparatory cell culture and cell seeding. However, investigators were blinded during the generation/analysis of the experimental data.

Reporting for specific materials, systems and methods

We require information from authors about some types of materials, experimental systems and methods used in many studies. Here, indicate whether each material, system or method listed is relevant to your study. If you are not sure if a list item applies to your research, read the appropriate section before selecting a response.

Materials & experimental systems

n/a	Involved in the study	
☐	☒	Antibodies
☐	☒	Eukaryotic cell lines
☒	☐	Palaeontology
☐	☒	Animals and other organisms
☐	☒	Human research participants
☒	☐	Clinical data

Methods

n/a	Involved in the study	
☒	☐	ChIP-seq
☒	☐	Flow cytometry
☒	☐	MRI-based neuroimaging

Antibodies

Antibodies used

4EBP1 Cell signaling 9452
LAMP2 Abcam 13524
LARS Cell signaling 13868
LC3b Novus Biologicals NB100-222
mTOR Cell signaling 2983
P62 Sigma-Aldrich P0067
p-AktSer473 Cell signaling 4060
PAN-leucylation Home made
p-mTORSer2448 Cell signaling 5536
p-S6K1Thr389 Cell signaling 9234
p-RPS6Ser235/236 Cell signaling 2211
p-SAPK/JNKthr183/tyr185 Cell signaling 9251
p-TSC2Ser1387 Cell signaling 5584
Puromycin Merk-millipore MABE343
RagA 142 leucylation Home made
SESTRIN2 Lubio 10795-1-AP
BA-F8 (MHCI) Developmental Studies Hybridoma Bank BA-F8
SC-71 (MHCIIa) Developmental Studies Hybridoma Bank SC-71
BF-F3 (MHCIIb) Developmental Studies Hybridoma Bank BF-F3
CD31-Alexa488 Biolegend 102414

	CD45-Alexa488 Biolegend 103122<br>α7 INTEGRIN-PE R&D Systems FAB3518P<br>SCAI-APC Biolegend 10811<br>PHD1 Novus Biologicals NB100-310<br>p-4EBP1Ser65 Cell Signaling 9451<br>SESTRIN1 Proteintech 21668-1-AP
Validation	Positive controls were used to validate specifiy

Eukaryotic cell lines

Policy information about cell lines

Cell line source(s)	Deutche Sammlung von Mikroorganismen und Zellkulturen (DSMZ)
Authentication	None of the cell lines were authenticated
Mycoplasma contamination	All cells were free from mycoplasma. Confirmed by testing every 2 weeks
Commonly misidentified lines (See ICLAC register)	We haven't used any misidentified cell lines.

Animals and other organisms

Policy information about studies involving animals; ARRIVE guidelines recommended for reporting animal research

Laboratory animals	Phd1 knockout (PHD1KO) mice (50% Swiss/ 50% 129S1 background)<br>HSA.iCre x RosamTmG (C57BL6/J background)<br>phd1 fl/fl x HSA mice (C57BL6/J background)
Wild animals	Study did not involve wild animals
Field-collected samples	Study did not involve samples obtained from the field
Ethics oversight	All animal procedures were approved by the Veterinary office of the Canton of Zürich, Switzerland (licence nr. ZH255-16) and by the local ethics committee of the KU Leuven, Belgium (P174-2014)

Note that full information on the approval of the study protocol must also be provided in the manuscript.

Human research participants

Policy information about studies involving human research participants

Population characteristics	Aged (n=8, 4 women and 4 men, 72.6 y ± 2.3 y) and young (n=8, 4 women and 4 men, 26.1 y ± 1.1 y) volunteers were recruited
Recruitment	Participants were recruited by worth of mouth and over the internet. The manner of recruitment has no impact on the results presented in the manuscript.
Ethics oversight	Ethical approval was obtained through the NHS Black Country Research Ethics Committee (13/WM/0429).

Note that full information on the approval of the study protocol must also be provided in the manuscript.
